# Trends in Socioeconomic Inequalities in the Prevalence of Chronic Non-Communicable Diseases in China: Evidence from Shaanxi Province During 2003–2013

**DOI:** 10.3390/healthcare13020178

**Published:** 2025-01-17

**Authors:** Dan Wang, Rashed Nawaz, Xiaojing Fan, Chi Shen, Sha Lai, Zhongliang Zhou, Jianmin Gao

**Affiliations:** 1 School of Public Policy and Administration, Xi’an Jiaotong University, Xi’an 710049, China; xjtuwangdan@stu.xjtu.edu.cn (D.W.); fanxj112@xjtu.edu.cn (X.F.); chi.shen@xjtu.edu.cn (C.S.); laisha@xjtu.edu.cn (S.L.); zzliang1981@xjtu.edu.cn (Z.Z.); 2 School of Public Health and Health Nutrition, Luohe Medical College, No.148, Daxue Road, Yuanhui District, Luohe 462002, China

**Keywords:** chronic non-communicable diseases, socioeconomic, inequality, concentration index, China

## Abstract

**Background**: The link between chronic non-communicable diseases (NCDs) and poverty in underdeveloped countries is debated. This study aims to examine socioeconomic inequalities related to NCDs and assess the contributing factors to these disparities. **Methods**: The study utilized data from the National Health Services Survey in Shaanxi Province for 2003, 2008, and 2013, having 71,766 respondents. The concentration index (CI) was employed to rigorously quantify the degree of socioeconomic inequality in the prevalence of non-communicable diseases (NCDs). The CI decomposition identified the contribution of each variable, while the horizontal inequity (HI) index was calculated annually to assess changes in inequality. Additionally, a Probit model was employed to examine the significant determinants contributing to the occurrence of NCDs. **Results**: The results show a significant increase in NCD prevalence with age, particularly for individuals aged 60 and above, who experienced a 286.55% rise from 2003 to 2013. Higher education levels are associated with decreased NCD prevalence, as evidenced by a 74.13% reduction for those with high school education or above. Additionally, wealthier individuals had a 15.31% lower prevalence of NCDs, indicating that higher socioeconomic status correlates with a reduced likelihood of chronic diseases. **Conclusions**: The study finds that NCD prevalence significantly increases with age, while higher education levels and greater wealth are associated with reduced prevalence. These findings highlight the need to target older populations and lower socioeconomic groups for effective NCD prevention and management. Policies should focus on improving educational opportunities and socioeconomic conditions to reduce the burden of NCDs, particularly among older and economically disadvantaged groups.

## 1. Background

Chronic non-communicable diseases (NCDs) are nowadays a key public health concern that poses significant health and developmental challenges worldwide, disproportionately affecting inhabitants in low- and middle-income countries (LMICs) [1,2,3]. Research in high-income countries shows that socioeconomic inequality in NCD prevalence contributes significantly to life expectancy and mortality gaps between the rich and poor. The rising burden of NCDs also poses serious economic and public health challenges, particularly in low- and middle-income countries [4,5,6]. NCDs are responsible for 71% of worldwide deaths, or 41 million deaths yearly. Of these, cardiovascular diseases cause 17.5 million deaths (46.2%), while diabetes accountable for 1.5 million (4%). Even though they are often connected to richer and elderly populations, NCDs excessively affect low- and middle-income countries (LMICs), where stressed healthcare systems worsen the issue [7,8,9,10,11]. China has experienced an increasing burden of single (65%) and multiple (54%) NCDs in Chinese inhabitants, attributed to economic growth and an aging population, accounting for 87% of the total deaths in the year 2019 [8]. In China, despite rising life expectancy, health inequalities persist due to widening income disparities, making it essential for policymakers to assess and address health gaps between socioeconomic groups with respect to NCDs [12,13,14].

Previous research has demonstrated that socioeconomic status is a key determinant of the distribution of chronic diseases in populations [15,16]. Moreover, health inequities arise from varying social conditions among different population groups, with socioeconomic status (SES) being a key factor in the distribution of NCDs [17]. Additionally, the relationship between SES and NCDs has shifted over time, and varies by region. Once more common among higher socioeconomic groups, NCDs are increasingly prevalent among the most disadvantaged populations [18]. A comparative study of socioeconomic status and the prevalence of NCDs across eight European countries found that most NCDs are more common among those with lower education levels. However, the relationship between NCD prevalence and economic status varies significantly across different types of diseases and countries, influenced by countries’ stage of socioeconomic development and health policies [19,20]. Studies in China have shown a positive correlation between higher socioeconomic status and the prevalence of hypertension and diabetes [21,22].

China has experienced a rapid epidemiologic shift from infectious diseases to non-communicable diseases, driven by an aging population, rapid domestic lifestyle changes, rapid economic growth, and urbanization [17]. The Chinese government recognized these challenges and initiated healthcare system reforms in 2009, marking a significant step towards addressing health inequalities [18]. The government aimed to strengthen the healthcare system by focusing on primary care and promoting equity in diagnosis and treatment. In 2009, a basic public health package was introduced to improve disease prevention and care for vulnerable groups, with funding increasing from 15 Yuan per person to 35 Yuan by 2013 [18]. This package included health records, education, geriatric care, and chronic disease management, alongside effective NCD prevention and control measures. However, the mechanisms by which socioeconomic inequality contributes to the rise in NCDs over time remain unclear, particularly in China’s less advanced western regions, like Shaanxi, where the shifting distribution of risk factors among different socioeconomic subgroups requires further investigation.

The association between NCDs and socioeconomic status can be examined in two ways: firstly, by examining the prevalence of NCDs, and secondly, by investigating the distribution of risk factors, like body mass index (BMI) and smoking, that are associated with the wealth index. NCDs are influenced by both unavoidable risk factors, such as age, and modifiable behavioral risks, including tobacco use and excessive alcohol consumption, which contribute to conditions like obesity and raised blood pressure, ultimately leading to disease [23]. Societal disparities in these risk factors contribute to more than half of the disparities in key NCDs [24,25,26]. The focus on NCD risk factors in advanced nations often highlights disparities that primarily affect the deprived. However, emerging nations, such as China, show varying patterns depending on the specific risk factors and their epidemiological context. In China, there is a limited understanding of how economic status influences NCD-related inequalities and their progression. Due to diverse societal structures, economic conditions, and lifestyles across regions, there are significant variations in the epidemiology of NCDs. This research focuses on Shaanxi Province, where healthcare resources are comparatively limited, and both economic and social development lag behind other regions such as Central and Eastern China. To our knowledge, this study is the first to explore changes in the relationship between socioeconomic inequality and NCDs using a comprehensive and representative sample of the general population.

The objective of this research is to address the gap in understanding socioeconomic-related disparities and horizontal equity in NCDs by analyzing household survey data from Shaanxi Province, China. We specifically examine the economic distribution of NCD prevalence, including smoking, drinking, and obesity, in Western China. By monitoring these inequalities, this study offers essential insights into socioeconomic disparities in NCD prevalence and risk factors, informing targeted public health strategies.

## 2. Materials and Methods

### 2.1. Data

The current research utilized data from the 3rd, 4th, and 5th National Health Services Surveys (NHSSs), along with extended samples from surveys conducted in Shaanxi Province in 2003, 2008, and 2013. The NHSS has been conducted every five years since 1983, coordinated and supervised by the Center for Health Statistics and Information of the National Health and Family Planning Commission of the People’s Republic of China. A multistage stratified cluster random sampling technique was utilized to collect representative samples in Shaanxi province [27]. First, the population was stratified into urban and rural areas. Next, counties (rural) and districts (urban) were randomly selected within each stratum. In the second stage, villages or residential communities were randomly chosen based on population size. Finally, systematic random sampling was applied to select households. This detailed sampling process ensured comprehensive coverage, high response rates (>85%), and minimized potential biases, enhancing data reliability. The NHSS questionnaire mostly includes sociodemographic characteristics (such as age, sex, schooling, household wealth, or expenses for the previous year), health status (e.g., the occurrence of NCDs), and behavior characteristics (e.g., tobacco and alcohol). For this research, face-to-face interviews were conducted by trained local healthcare workers, supervised by senior professionals, to ensure data consistency. Respondents were notified in advance, and up to three follow-up visits were made to maximize participation. Consistency checks on 5% of households, with re-surveys for missing or inconsistent responses, ensured over 95% agreement and data accuracy. The anonymized cross-sectional data were provided with the help of the Shaanxi Health and Family Planning Commission (www.sxhealth.gov.cn, accessed on 25 December 2024). The final sample consisted of 71,766 respondents. According to the Shaanxi 2017 statistical yearbook, the overall population of Shaanxi Province was 36.7 million in 2003, 37.2 million in 2008, and 37.6 million in 2013 [28]. Moreover, self-reported smoking and alcohol use may be biased by income, with lower-income individuals under-reporting and wealthier individuals over-reporting due to social desirability and health literacy. Behaviors were analyzed separately, although socioeconomic interactions may influence NCD disparities. To address these limitations, the study implemented standardized questionnaires, interviewer training, and validation subsamples (5% of the sample with >95% agreement). Ethical approval was reviewed and granted by the Ethics Committee, Health Science Center, Xi’an Jiaotong University. All the respondents of this study were aged 18 years or older, and were eligible to fill out the questionnaire regarding the occurrence of NCDs.

### 2.2. Measurements of the Prevalence of NCDs

The primary focus of the current study was physician-diagnosed NCDs within the last 6 months. In the NHSS, the NCDs were categorized as either (1) NCDs diagnosed within the previous months, or (2) NCDs in patients who had been continuously receiving treatment since the disease was first diagnosed, and for whom the treatment had continued through the last six months or more. The NHPC coded the diagnosed NCDs according to the 10th revision coding system protocols of the International Classification of Diseases (IDC) [28], which serves as the international standard of diagnostic classification for clinical practice and general epidemiological and health management.

### 2.3. Behavior

The selection of smoking, alcohol consumption, and BMI (body mass index) was based on their well-documented association with NCDs and their modifiability through public health interventions. These behaviors are recognized as key determinants of NCD prevalence in global and national studies [23]. The categorization of these risks was designed to capture relevant population-level differences while maintaining consistency with established guidelines. In the current research, alcohol consumption was characterized into two complete clusters: harmful use (weekly or more frequent) vs. non-drinkers. The harmful use thresholds reflect guidelines from WHO and the Chinese Ministry of Health. These categories distinguish between those at negligible risk and those at elevated risk for alcohol-related health outcomes [23,28]. Overweight was defined as having a BMI of 24 kg/m^2^ or more; these thresholds are specific to the Chinese population, where lower BMI levels are associated with elevated metabolic risks compared to Western populations. Furthermore, individuals with a BMI of 28 kg/m^2^ or more were classified as obese [23].

Self-reported behavioral risks may be biased by income, with under-reporting among lower-income groups and over-reporting among wealthier individuals. To address this, standardized questionnaires, interviewer training, and a 5% validation subsample (showing >95% consistency) were employed.

### 2.4. Economic Status

The information on yearly household wealth and expenses was available from the NHSS. As per O’Donnell et al., economic status is defined based on the household consumption expenses reported by individuals, and is adjusted by taking into account the number of adults in each household [29]. These expenses were then further categorized into five percentiles; the first quintile represented the poorest income group, whereas the fifth quintile represented the richest. It is proposed that self-stated expenses are improved indicators of household economic status, especially in emerging nations.

### 2.5. Concentration Index

The concentration index (CI) was employed to compute the extent of income-related disparity. The CI ranges from −1 to +1, with 0 highlighting no income-related disparity. A positive (negative) CI indicates a pro-rich (pro-poor) disparity in health. The CI was calculated utilizing Equation (1):

(1) $$C=\frac{2}{\mu }\mathrm{cov}(y,r)$$

where *C* demonstrates the concentration index, *y* is the health index, *μ* is the mean of the health index, and *r* is the fractional income rank, ranging from 0 to 1. The rank of the individual *i* is written as *r_i_* = *i/N*, in which *N* is the number of individuals.

The standard deviation of the CI was calculated using Equation (2):

(2) $$\mathrm{var}(\hat{C})=\frac{1}{n}[\frac{1}{n}\sum_{i=1}^{n}a_{i}^{2}-{\left(1+C\right)}^{2}]$$

in which $a_{i}=\frac{h_{i}}{\mu }(2r_{i}-1-C)+2-q_{i-1}-q_{i}$ and $q_{i}=\frac{1}{\mu n}\sum_{j=1}^{i}h_{j}$.

In this study, the CI provides a quantifiable measure of socioeconomic inequality in NCD prevalence. A positive CI indicates that NCDs are more prevalent among wealthier groups, while a negative CI shows that poorer groups bear a disproportionate burden. Over time, the shift in CI is critical for understanding how epidemiological transitions and systemic disparities in healthcare access and behavioral risks have exacerbated health inequalities. Therefore, the CI not only captures the extent of inequality, but also serves as a diagnostic tool to guide public health strategies.

### 2.6. Decomposition of CI

The decomposition of the CI was conducted to highlight the strength of contributory variables to health disparity, based on a regression framework (see Equations (3) and (4)).

(3) $$y_{i}=\alpha +\sum_{j}\beta_{j}^{m}x_{ji}+\sum_{k}\gamma_{k}^{n}z_{ki}+\varepsilon_{i}$$

where $y_{i}$ represents the prevalence of NCDs, and *x* highlights inevitable health factors, including sex and age, whereas *y* refers to preventable well-being factors, such as self-reported yearly individual expenses, marital status, and schooling [30], and behavioral risk factors, including use of tobacco, harmful alcohol consumption, and BMI $\beta_{j}^{m}$ The bordering impact of every factor assessed for the sample constitutes the error term. the following equation explains the decomposition of the concentration index *C*:

(4) $$C=\sum_{j}(\beta_{j}^{m}{\bar{x}}_{j}/\bar{y})C_{j}+GC_{u}/\bar{y}$$

where $\bar{y}$ represents the mean of *y*, *C_j_* symbolizes the concentration index for *x_j_*, and ${\bar{x}}_{j}$ represents the mean of *x_j_*. The first term on the right side signifies the effect of independent factors on disparity, whereas the latter term is generalized as the concentration index $\varepsilon_{i}$.

Decomposing the CI results into economic quantiles and behavior-related risks provides actionable insights into the drivers of inequality, enabling more targeted and effective interventions. Economic quantile decomposition highlights the increasing NCD burden on poorer populations, guiding resource allocation toward the most vulnerable groups. Behavioral risk decomposition identifies modifiable contributors, such as smoking and obesity, which disproportionately affect low-income populations. This approach not only quantifies the relative contributions of socioeconomic and behavioral factors, but also tracks changes over time, offering critical insights for designing effective, equity-focused public health strategies.

### 2.7. Measurement of Health Inequity

The horizontal inequity (HI) in health was measured by utilizing the outcomes of the decomposition analysis. HI designates the health disparity among individuals with similar unavoidable health circumstances [30]. In this research, sex and age were considered unavoidable health variables, the HI index of NCD prevalence was calculated by subtracting the contributions of sex and age from the CI of NCD prevalence, as shown in Equation (4). All analyses were performed using Stata version 15 (Stata Corp LP, College Station, TX, USA),while visualizations were created in Python version 3.11.10 to ensure reproducibility and clarity of results.

## 3. Results

A total of 9976 residents in 2003, 14,628 in 2008, and 47,162 in 2013 were included in the current study. Table 1 illustrates a comprehensive description of the sociodemographic features. In 2003, 13.05% of the respondents reported having NCDs, increasing to 20.02% in 2008 and 23.54% in 2013. Moreover, the percentage of the respondents who smoked often or every day decreased slightly from 30.02% to 29.86%, but this decrease in outcomes was not statistically significant. Concerning consumption of alcohol, the proportion of respondents who drank more than once a week reduced from 19.03% to 9.08% over the investigated decade. Furthermore, in 2013, 23.67% of the respondents significantly reported being overweight/obese.

### 3.1. Distribution of NCD Prevalence and NCD Risk Factors

Table 2 demonstrates the distribution of NCD prevalence, behavior-related risks, and overweight/obesity across different economic quantiles in remote western areas of China. For the years 2003 and 2008, NCDs were more commonly reported among the richer respondents as compared to the poorer respondents (from the poorest to the richest, in 2003: 12.40%, 11.51%, 11.09%, 13.87%, 16.26%; and in 2008: 19.19%, 18.53%, 18.20%, 20.38%, 23.98%) respectively. However, in 2013, the poorer respondents reported higher NCDs (from the poorest to the richest: 26.98%, 20.73%, 21.22%, 22.42%, and 25.54%).

Concerning behavior-related risks and overweight/obesity, smoking was more popular among richer respondents in 2003, compared to 2008 and 2013. Alcohol consumption was also more prevalent among the richest respondents over the decade, and overweight/obesity was significantly higher among the richest population group in 2013. In summary, our results demonstrated that the richest population groups were more likely to suffer from NCDs compared to the poor population groups in 2003 and 2008 in Shaanxi Province, China. Nevertheless, in 2013, a greater percentage of NCDs cases were reported among the poorer group of the population as compared to the richest population group.

### 3.2. Concentration Index (CI)

The concentration index (CI) was used to measure the disparities in NCD prevalence and its risk factors across economic quantiles in Shaanxi province (Table 2). The CI for the prevalence of NCDs was changed from 0.0571 to −0.0143 (*p* < 0.001; 95% CI), indicating a significant change. Smoking was identified as negatively significant for the respondents in 2013, whereas drinking was found to be more popular among the richest group of the population over the decade. These outcomes of our study indicated that the association and inequalities of NCD prevalence have reversed over time, changing from a greater occurrence in the richest socioeconomic cluster to a greater occurrence among the most disadvantaged group of the residents. We expanded the analysis of confidence intervals (CIs) by decomposing the results according to economic quantiles (poorest, poorer, middle, richer, richest) and behavior-related risks (such as smoking and alcohol consumption). This decomposition allows for a more detailed exploration of the variability in NCD risks across different socioeconomic groups. For example, in lower-income groups, the CI for the relationship between smoking and cardiovascular disease was wider, indicating greater uncertainty or variability in the impact of smoking within this group compared to higher-income groups. These findings suggest that socioeconomic factors may modulate the impact of risk behaviors on NCD outcomes.

### 3.3. Decomposition of the Concentration Index

Inequalities in NCD prevalence were additionally measured by decomposing the concentration indices into their determined components. Table 3 demonstrates a detailed decomposition of the CIs for the prevalence of NCDs. For each subsample, the CI and marginal impact were measured based on the Probit regression approximations, presenting the contribution to the NCD prevalence disparities and the percentage contribution of each determined component. Our results found that, in 2003 and 2008, economic status was the primary contributor to NCDs disparities. In 2003, the richest quantile contributed 56.85% to the total disparity, and respondents above 60 were responsible for 42.04%. In 2008, the richest quantile contributed 62.61%, whereas the respondents aged 45–59 were responsible for 58.02%. By the year 2013, the inequalities in NCD prevalence were mainly explained by the age group of 45 to 59 or those above 60 years, contributing 150.65% and 286.55%, respectively. Among all the contributors, two contributors in 2013, age and economic status, were highlighted for their constructive contributions towards greater pro-poor inequality, indicating that NCDs had become more prevalent among the poorer population group compared to the richest population group.

### 3.4. Subgroup Analyses

We examined the distribution of NCD burden among low-educated older individuals across income quintiles, finding that those in the lowest quintiles significantly contribute to the negative concentration index, highlighting socioeconomic health disparities. We also explored behavioral risk factors (e.g., smoking, drinking) within this subgroup to identify potential mechanisms behind these inequalities. Table 4 presents the decomposition of the concentration index (CI) by various subgroups, for individuals aged 60 and above, across the years 2003, 2008, and 2013. The table shows both absolute changes (dy/dx) and relative percentage changes. For the income groups, richer and richest exhibited substantial positive concentrations, particularly in 2003 and 2008, with richer having a dy/dx of 0.036 (39.53%) increases in 2003, and richest showing a significant rise in 2008 (dy/dx = 0.082, 129.32%). However, both groups saw a sharp decline in CI by 2013 (richer: dy/dx = −0.017, −43.09%; richest: dy/dx = 0.014, −63.09%). In contrast, the poorest group experienced a notable reduction in inequality, with dy/dx dropping to −0.038 in 2013, reflecting a −67.34% decrease. Married individuals saw an increase in concentration, with a significant rise from dy/dx = 0.111 (4.32%) in 2003 to 0.177 (84.73% decrease) in 2013. Educational attainment played a serious part, with high school and above showing a large increase in concentration, where the CI dropped from −0.103 (118.6%) decrease in 2003 to −0.153 (242.12%) increase in 2013. Lifestyle factors such as smoking and drinking exhibited varying impacts, with smoking seeing a reduction in concentration (dy/dx = −0.038, −4.67%) in 2013 and drinking showing a moderate decrease in inequality over time (dy/dx = −0.049, 8.98% in 2013). Overall, the decomposition reveals shifting trends in inequality, with some subgroups (like the wealthier, married, and more educated) experiencing increasing concentration, while others (especially the poorer and those with lower education) saw improvements in reducing inequality by 2013.

#### 3.4.1. NCD Prevalence by Education Level and Income Group (2003, 2008, 2013, Age ≥ 60)

**Figure 1:**
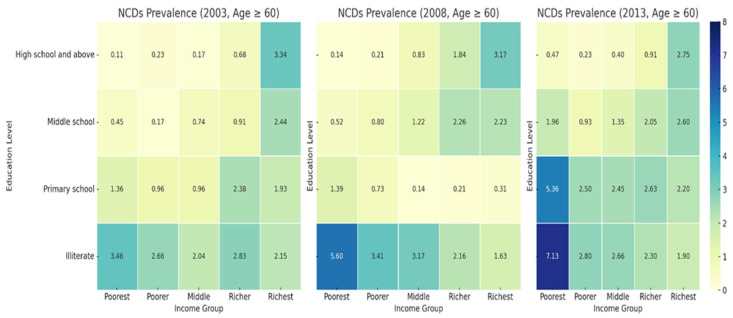
Prevalence of NCDs among those Age ≥ 60 from 2003–2013.

The heat maps indicate a clear socioeconomic and educational gradient in smoking prevalence among individuals aged 60 and above in Figure 1. Across all years, smoking prevalence is highest in the poorest illiterate group, rising from 3.46% in 2003 to 5.60% in 2008, and peaking at 7.13% in 2013. In contrast, the richest illiterate group shows a much lower prevalence, decreasing from 2.15% in 2003 to 1.900% in 2013. The data show that while prevalence slightly decreased in some groups, smoking remained disproportionately high in low-income, less-educated populations.

#### 3.4.2. Prevalence of Smoking Among Age ≥ 60 from 2003 to 2013

**Figure 2:**
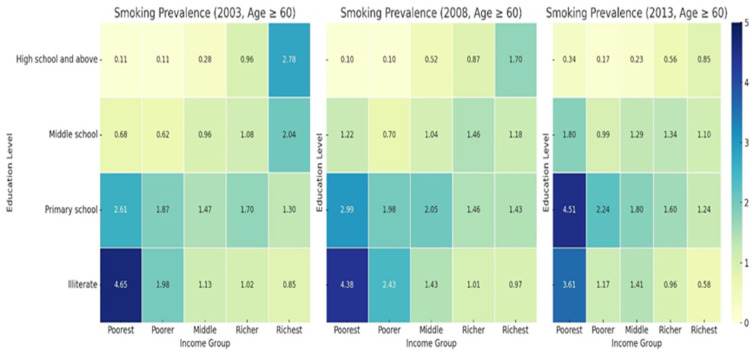
Prevalence of smoking among those Age ≥ 60 from 2003 to 2013.

Figure 2 illustrates heatmaps showing a persistent socioeconomic and educational gradient in smoking prevalence among individuals aged 60 and above. Smoking rates are highest in the poorest illiterate group, with values of 4.65%, 4.38%, and 3.61% in 2003, 2008, and 2013, respectively. In contrast, the richest illiterate group consistently exhibits lower smoking prevalence, declining from 0.85% in 2003 to 0.58% in 2013.

Smoking prevalence remains low among individuals with high school education or above, consistently below 1.53% across all income levels and years. Among middle school and primary school groups, smoking prevalence decreases as income increases, indicating an inverse relationship between socioeconomic status and smoking. These results underscore the need for targeted tobacco control policies aimed at low-income, less-educated populations.

#### 3.4.3. Drinking Prevalence by Education Level and Income Group Age ≥ 60 (2003, 2008, 2013)

**Figure 3:**
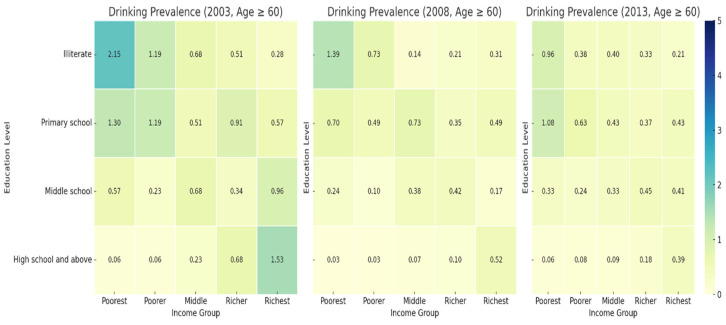
Prevalence of drinking among those Age ≥ 60 from 2003 to 2013.

Figure 3 shows that the drinking prevalence follows a similar pattern, with the highest rates among illiterate individuals in the poorest group (2.15% in 2003, decreasing to 0.96% in 2013). In contrast, the richest illiterate group had minimal drinking prevalence throughout the years, remaining below 0.5%. Among individuals with primary school education, the prevalence ranged from 1.30% (poorest group) to 0.37% (richer group) by 2013. Drinking rates among those with middle school education and high school education were consistently low across all income groups.

Smoking and drinking behaviors, as well as NCD prevalence, show a clear socioeconomic and educational gradient. In 2013, the smoking prevalence among the poorest illiterate group was 3.61%, while the NCD prevalence reached 7.13%, highlighting significant disparities. The NCD prevalence steadily increased across all income groups. The poorest group saw the sharpest rise, with the NCD prevalence increased from 3.46% in 2003 to 7.13% in 2013. These findings call for targeted interventions focusing on low-income, less-educated populations.

#### 3.4.4. Temporal Trends in NCD Prevalence by Economic Status (Illiterate, Age ≥ 60)

**Figure 4:**
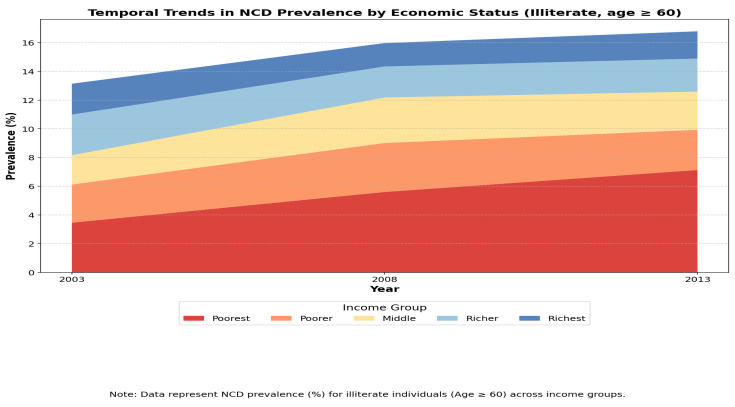
Temporal trends in NCD Prevalence by education level and economic status (age ≥ 60).

The stacked area chart in Figure 4 shows a steady rise in NCD prevalence across all income groups from 2003 to 2013. In 2003, the NCD prevalence in the poorest group was 3.46%, increasing to 7.13% by 2013, while in the richest group, it decreased from 5.2% to 1.91%. This indicates a widening gap in health outcomes, with lower-income groups experiencing a faster increase in NCD prevalence compared to wealthier groups.

### 3.5. Horizontal Inequity

The horizontal inequity (HI) of health status was measured by subtracting the contributions of demographic characteristics (i.e., age and sex) from the CI of the prevalence of NCDs. Table 5 highlights that the HI indices were 0.017, 0.036, and 0.005 for 2003, 2008, and 2013. In 2003, the total contribution of observable factors was 101.58%, highlighting that −1.58% of the disparity in prevalence was described by the regression error term. Additionally, in 2008, the total contribution of observables was 100.85%, meaning that −0.86% of the disparity was also defined by the regression error term. Furthermore, the prevalence of NCDs was primarily explained by the respondents aged 45 and above (134.81%) in 2013. The entire contribution of the observables was 91.15%, showing that 8.85% of the negative contribution to the disparity was attributed to the error term of the regression. The horizontal inequity (HI) index quantifies socioeconomic inequities in NCD prevalence after adjusting for unavoidable variables such as age and gender. Across the study period (2003–2013), the HI decreased from 0.017 in 2003 to 0.005 in 2013, suggesting a narrowing of horizontal inequities over time. This reduction is consistent with a shift in the NCD burden from wealthier to poorer populations, as evidenced by corresponding changes in the concentration index (CI).

## 4. Discussion

The current research examined the prevalence of socioeconomic inequalities in NCDs and their trends among different economic quantiles in China for the years 2003, 2008, and 2013. Additionally, this article expands the literature by decomposing the disparity in NCD prevalence and investigating its horizontal equity in China. The large-scale demonstrative household survey data reveal a shift in NCD prevalence from being higher among the wealthy to disproportionately affecting the poor in China. Furthermore, economic status and age are identified as key factors explaining this trend of inequity.

This study reveals a shift in NCD prevalence in China, from being higher among the richest to excessively affecting the poor, especially aging individuals, who are at higher risk of NCDs. These findings of our study corroborate those of prior published studies. Several studies on middle-income countries have also demonstrated significant variations in NCD prevalence and inequalities [30]. This is mainly because of the nation’s economic development phase and its well-being strategies and procedures. Over the past three decades, China has experienced momentous economic development. In the year 2009, China began to balance the primary public healthcare services 2009, yielding appropriate management of NCDs Despite the hard work, the increase in the occurrence of NCDs nationwide has not been prevented [31]. Given the distinctiveness of societal infrastructures, financial profiles, and lifestyles among different regions, inequalities in the epidemiological characteristics of NCDs are likely to exist among diverse population groups and areas [18,27,31]. The present study precisely examines the situation of Shaanxi Province, China, where healthcare resources are comparatively lacking, and both monetary and societal growth lag behind the eastern and central parts of China. Individuals in economically underdeveloped regions, especially those with low socioeconomic status face difficulties in acquiring timely and adequate diagnosis and treatment of NCDs compared to populations residing in developed regions or with greater socioeconomic positions. Additionally, this study also quantitatively calculates the population distribution of NCDs using a central index, further confirmed by factors related to NCD prevalence [32,33]. Precisely, the study focuses on smoking, alcohol consumption, and obesity. The findings indicate that alcohol consumption and obesity are more concentrated and significant among the rich, while the distribution of smoking in the population has shifted from the rich to the poor.

It is of great importance to highlight that the greater occurrence of NCDs among the rich can be attributed to their greater likelihood of receiving a diagnosis compared to those with lower incomes. However, since 2009, China has made efforts to equalize basic public health services, including NCD management. Additionally, previous studies have indicated no significant difference between self-reported and measured hypertension in the Chinese population [34,35], which reduces the potential bias of self-reported NCD data in our study. Regarding harmful alcohol use, we found that frequent drinking, defined as more than once per week, was concentrated among the wealthy population. Remarkably, individuals who drink less may be more susceptible to NCDs, possibly due to some individuals who stop drinking after being diagnosed with an NCD. There is a well-established connection between the consumption of alcohol and NCDs, supporting the WHO’s call for evidence-based strategies to reduce harmful alcohol use [19,34,36]. Concerning smoking, the prevalence has shifted from the rich to the poor group of the population. In 2013, the low socioeconomic clusters revealed a significantly greater rate of tobacco use compared to high socioeconomic clusters [37]. This is consistent with the worldwide trend of tobacco consumption, where smoking follows an inverted U-shape; as a country develops, the ratio of smoking initially inclines among poorer individuals, but begins to decline among rich population groups on a specific economic development level [38].

Numerous studies regarding obesity have suggested that as countries develop, the burden of deprived well-being behaviors shifts from the rich to the less wealthy population. For example, a prior study highlighted that in the southeastern part of Brazil, the prevalence of obesity among females had moved from the richest quartile to the deprived quartile since 1975 [39]. Table 3 demonstrates that BMI is one of the two most significant contributors to the CI, indicating that the rich population group faces a greater issue with overweight/obesity problems compared to the less rich counterparts [40].

While comparing our results with Chinese data from the literature, we found similar positive CIs for the prevalence of NCDs [18]. Our study suggests that rich individuals reported a greater occurrence of NCDs in 2008. However, this trend has changed over time with the development of the social economy in China. Our findings by 2013 are consistent with studies from developed countries, such as Denmark [41] and Canada [42,43]. Our study demonstrates that age and socioeconomic status are two key factors that influence these trends. Decomposing the CI by economic quantiles and behavior-related risks enhances our understanding of the interaction between income and health behaviors in the context of NCDs. Our findings suggest that the association between smoking and cardiovascular disease varies more in lower-income groups, indicating that socioeconomic factors may impact the way behavioral risks contribute to NCD outcomes. Additionally, these outcomes suggest that targeted programs, such as subsidized smoking cessation and affordable nutrition education, can directly address cost barriers and health literacy gaps, thereby reducing NCD risks. Regular screenings, mobile clinics, and community-based medication adherence efforts are vital where healthcare access is limited, helping to lower morbidity and associated costs. Future studies could explore how specific economic interventions, such as school-based health education and community literacy initiatives, empower individuals to adopt healthier behaviors and enhance long-term socioeconomic prospects, ultimately reducing NCD disparities.

This study has several limitations. Its regional focus on Shaanxi Province may limit the generalizability of the findings across China, particularly to more developed eastern regions, and urban–rural disparities in healthcare access and quality may influence NCD trends differently. Additionally, unobservable factors, such as genetic and environmental influences, could affect NCD prevalence and introduce bias in the estimates. The research explores associations rather than causality, as factors like income and well-being may mutually influence each other. Environmental factors, such as air pollution and pesticide exposure, as well as socioeconomic determinants, like low health literacy, delayed care-seeking, and lifestyle changes driven by urbanization and income inequality, also contribute to these disparities. Future research should include diverse regions to better understand the full scope of NCD disparities, integrate environmental and healthcare data, and explore behavioral and cultural influences on risk factors. Longitudinal studies are necessary to establish causal links between socioeconomic status, healthcare access, and NCD outcomes, and to inform targeted interventions.

## 5. Policy Implications

The outcomes of our research highlight key policy implications for addressing the growing socioeconomic inequalities in NCD prevalence in China. Policymakers should prioritize targeted interventions to reduce the NCD burden among lower-income and aging populations, focusing on improving healthcare access, promoting healthy behaviors, and addressing broader social determinants of health. Strengthening primary healthcare is essential, with efforts aimed at expanding rural healthcare infrastructure and increasing access to affordable diagnostic and treatment services for NCDs. This would ensure that underserved populations receive timely and effective care in rural and underdeveloped areas. Behavioral interventions should target key risk factors, like smoking and unhealthy diets, through tailored programs. Smoking cessation efforts should focus on lower-income and rural populations, while public education campaigns can promote healthier eating. Community-based programs can raise awareness and provide ongoing monitoring of at-risk individuals. Public health campaigns should target modifiable risk factors, such as smoking, alcohol consumption, and obesity, with a focus on disadvantaged groups, while addressing obesity in wealthier populations. Strengthening health literacy through education and offering subsidized smoking cessation and nutrition programs can help to reduce disparities. Additionally, policies promoting healthy aging and equitable healthcare access across economic groups are essential for reducing the overall NCD burden.

## 6. Conclusions

The outcomes of this study propose that a significant shift has occurred in the prevalence of NCDs in China, with the burden gradually affecting the poorer group of the population, particularly among individuals aged above 60. Economic status and age were the important factors that led to the inequity. This could draw the attention of policymakers in two ways. Firstly, effective NCD prevention and management needs to be targeted at the elderly and groups with lower socioeconomic status. Secondly, the policies should focus on improving educational opportunities and social–economic conditions to reduce the social–economic inequity associated with NCDs.

## Figures and Tables

**Table 1:** Variable descriptions and descriptive analysis.

Variables	Variable Descriptions	N (%)			*p*
		2003	2008	2013	
Total observations		9976	14,628	47,162	
Prevalence of NCDs	Whether doctors have diagnosed NCDs or not	1302 (13.05)	2928 (20.02)	11,100 (23.54)	<0.001
Age group					
18–44	18 ≤ Age ≤ 44	5498 (55.11)	7071 (48.36)	19,423 (41.19)	<0.001
45–59	45 ≤ Age ≤ 59	2713 (27.20)	4675 (31.97)	15,749 (33.40)	
60 and above	60 ≤ Age	1765 (17.69)	2875 (19.66)	11,979 (25.41)	
Gender					
Male	Male = 1, else = 0	4974 (49.86)	7175 (49.07)	23,013 (48.80)	<0.001
Female	Female = 1, else = 0	5002 (50.14)	7446 (50.93)	24,149 (51.20)	
Economic quantiles					
Poorest	Poorest = 1, else = 0	1624 (20.12)	2400 (20.10)	9459 (20.06)	<0.001
Poorer	Poorer = 1, else = 0	1605 (19.89)	2385 (19.97)	9672 (20.51)	
Middle	Middle = 1, else = 0	1614 (20.00)	2413 (20.20)	9167 (19.44)	
Richer	Richer = 1, else = 0	1615 (20.01)	2359 (19.75)	9445 (20.03)	
Richest	Richest = 1, else = 0	1613 (19.99)	2386 (19.98)	9419 (19.97)	
Marital status					
Unmarried	Unmarried = 1, else = 0	1120 (11.35)	1847 (12.64)	4693 (9.95)	<0.001
Married	Married = 1, else = 0	8042 (81.50)	11,427 (78.20)	38,454 (81.56)	
Others	Divorce/Wisdom/Others = 1, else = 0	705 (7.15)	1339 (9.16)	4002 (8.49)	
Education status					
Illiterate	No formal education (illiterate) = 1, else = 0	1498 (5.19)	2150 (14.72)	7608 (16.14)	<0.001
Primary school	Primary school = 1, else =0	2045 (20.74)	2985 (20.44)	11,624 (24.65)	
Middle school	Middle school = 1, else =0	3760 (38.14)	5259 (36.02)	18,216 (38.63)	
High school and above	High school and above = 1, else = 0	2556 (25.93)	4208 (28.82)	9703 (20.58)	
Area					
Urban	Urban = 1, else = 0	5007 (50.19)	6683 (45.69)	17,372 (36.83)	<0.001
Rural	Rural = 1, else = 0	4969 (49.81)	7945 (54.31)	29,790 (63.17)	
Smoke					
No	Never smoke = 1, else = 0	6.878 (69.98)	10,267 (70.98)	33,037 (70.14)	0.118
Yes	Often smoke or smoke everyday = 1, else = 0	2951 (30.02)	4198 (29.02)	14,067 (29.86)	
Drink					
No	Less than one time a week = 1, else = 0	7867 (80.97)	12,727 (90.92)	37,828 (81.24)	<0.001
Yes	More than one time a week = 1, else = 0	1849 (19.03)	1271 (9.08)	4220 (9.08)	
BMI					
Underweight	BMI < 18.5 = 1, else = 0	--	---	4609 (9.79)	<0.001
Normal weight	18.5 ≤ BMI < 24 = 1, else = 0	--	--	31,333 (66.55)	
Overweight	24 ≤ BMI < 28 = 1, else = 0	--	--	9582 (20.35)	
Obesity	BMI ≥ 28 = 1, else = 0	--	--	1561 (3.32)	

**Table 2:** The CI of the prevalence of NCDs in different economic quantiles from 2003 to 2013 in China.

Variables	Year	Poorest	Poorer	Middle	Richer	Richest	Total	CI	SE of CI	95% CI
Prevalence of NCDs	2003 (%)	12.40	11.51	11.09	13.87	16.26	13.05	0.0571 c	0.0150	0.0278 0.0865
	2008 (%)	19.19	18.53	18.20	20.38	23.98	20.02	0.0489 c	0.0096	0.0302 0.0677
	2013 (%)	26.98	20.73	21.22	22.42	25.54	23.56	−0.0143 b	0.0049	−0.0238 −0.0048
Smoke	2003 (%)	27.52	28.33	29.92	34.19	31.81	30.34	0.0408 c	0.0098	0.0211 0.0596
	2008 (%)	30.57	29.77	27.63	28.60	28.56	29.03	−0.0123	0.0075	−0.0270 0.0024
	2013 (%)	30.37	30.89	30.58	29.92	28.06	29.98	−0.0139 c	0.0041	−0.0220 −0.0058
Drink	2003 (%)	16.69	19.17	19.38	19.10	22.44	19.32	0.0559 c	0.0133	0.0289 0.0811
	2008 (%)	9.11	8.89	8.54	8.54	10.45	9.09	0.0221	0.0155	−0.0082 0.0526
	2013 (%)	7.85	8.68	8.77	9.75	10.87	9.12	0.0685 c	0.0086	0.0515 0.0853
Overweight/obesity	2013 (%)	19.21	21.52	22.04	25.61	30.60	23.56	0.1037 c	0.0048	0.0880 0.1069
High school and above	2003 (%)	10.22	14.20	21.89	32.42	52.13	25.93	0.1130 c	0.0032	0.1071 0.1197
	2008 (%)	11.34	17.39	25.66	36.64	54.02	28.83	0.1094 c	0.0026	0.1043 0.1146
	2013 (%)	9.88	16.00	16.88	24.12	37.82	20.37	0.0910 c	0.0016	0.0884 0.0947
60 and above	2003 (%)	25.72	37.28	30.47	27.10	26.00	30.03	−0.0159	0.0245	−0.0641 0.0320
	2008 (%)	36.97	37.18	45.03	42.07	48.72	41.99	0.0598 c	0.0350	0.0814 0.2190
	2013 (%)	38.91	42.99	47.13	48.15	56.95	46.82	0.0757 c	0.0056	0.0647 0.0867
Rural	2003 (%)	9.99	11.47	11.29	12.99	14.35	12.02	0.0688 b	0.0240	0.0219 0.1159
	2008 (%)	18.61	19.50	15.52	20.45	19.43	18.70	0.0214	0.0159	−0.0094 0.0530
	2013 (%)	23.39	23.99	22.51	22.03	24.43	23.27	−0.0010	0.0061	−0.0129 0.0109

b, c: significantly different from zero at the 0.01 and 0.001 levels, respectively; CI: Concentration index.

**Table 3:** Decomposition of concentration indices of NCD prevalence.

Variables	2003			2008			2013		
	dy/dx	Con.	%	dy/dx	Con.	%	dy/dx	Con.	%
Age group									
45–59	0.130 c	0.015	26.75	0.209 c	0.028	58.02	0.215 c	0.022	−150.65
Age group 60 and above	0.261 c	0.024	42.04	0.351 c	−0.016	−32.38	0.408 c	−0.041	286.55
Female	0.024 c	0.001	0.94	0.016 b	0.000	0.35	0.013 b	0.000	−1.09
Poorer	0.001	−0.001	−1.20	0.009	−0.004	−7.34	−0.017 c	0.004	−30.22
Middle	−0.005	0.000	−0.20	0.003	0.000	0.00	−0.016 c	0.000	3.04
Richer	0.016	0.010	17.45	0.017	0.007	14.76	−0.010 a	−0.004	25.81
Richest	0.027 b	0.032	56.85	0.040 c	0.030	62.21	0.003	0.002	−15.31
Married	0.070 c	0.002	2.72	0.085 c	0.004	8.38	0.084 c	0.006	−39.90
Others	0.073 c	−0.003	−4.94	0.139 c	−0.004	−7.88	0.14 c	−0.005	35.64
Primary school	−0.005	0.002	2.75	0.018 a	−0.003	−5.13	−0.016 c	0.002	−13.01
Middle school	−0.025 c	0.002	3.49	−0.019 a	0.002	3.47	−0.046 c	−0.002	10.70
High school and above	−0.019 a	−0.013	−22.22	−0.034 c	−0.015	−30.20	−0.047 c	−0.011	74.13
Rural	0.01	−0.012	−21.34	−0.004 c	0.018	36.72	−0.009 b	0.003	−18.07
Smoke	0.011	−0.001	−2.52	−0.009	0.000	0.33	−0.027 c	0.000	−3.30
Drink	−0.030 c	0.001	1.03	−0.022 a	0.000	−0.44	−0.036 c	−0.001	6.60
18.5–24							−0.003	0.000	−0.78
24–28							0.098 c	0.008	−53.71
28 and above							0.213 c	0.004	−25.29

a, b, c: significantly different from zero at the 0.05, 0.01, and 0.001 levels, respectively; dy/dx means partial effects of each variable and evaluated at sample means; Con. means contribution to CI; % means percentage of contribution to CI.

**Table 4:** Decomposition of concentration index in subgroups (age >60).

Variables	2003			2008			2013		
	dy/dx	Con.	%	dy/dx	Con.	%	dy/dx	Con.	%
Female	−0.001	0	−0.05	−0.014	−0.0001	−0.3	−0.029	−0.0004	2.54
Poorer	0.002	−0.0012	−2.15	0.002	−0.0008	−1.72	−0.038	0.0096	−67.34
Middle	−0.0	0	0	0.009	0	0.01	−0.031	−0.0008	5.86
Richer	0.036 a	0.0226	39.53	0.035	0.0153	31.2	−0.017	−0.0062	43.09
Richest	0.072 a	0.0869	152.11	0.082	0.0632	129.32	0.014	0.009	−63.09
Married	0.111 a	0.0025	4.32	0.158	0.0076	15.6	0.177	0.0121	−84.73
Others	0.258 a	−0.01	−17.45	0.357	−0.0098	−20.14	0.41	−0.0148	103.42
Primary school	−0.045 a	0.0131	22.99	−0.033	0.0047	9.53	−0.066	0.0076	−52.86
Middle school	−0.109 a	0.0086	15.1	−0.131	0.0115	23.6	−0.162	−0.0054	37.93
High school and above	−0.103 a	−0.0677	−118.6	−0.154	−0.0665	−135.93	−0.153	−0.0346	242.12
Rural	−0.019 b	0.0235	41.17	−0.008	0.0374	76.44	−0.028	0.0082	−57.2
Smoke	0.013	−0.0018	−3.16	−0.007	0.0001	0.26	−0.038	0.0007	−4.67
Drink	−0.039 a	0.0008	1.32	−0.038	−0.0004	−0.78	−0.049	−0.0013	8.98
18.5–24							−0.025	0.0009	−6.18
24–28							0.07	0.0055	−38.6
28 and above							0.168	0.0028	−19.91

a, b: significantly different from zero at the 0.05, 0.01.

**Table 5:** Contributions of factors and horizontal inequity of NCD prevalence.

Variables	2003		2008		2013	
	Con.	%	Con.	%	Con.	%
Unavoidable variables	0.039	69.74	0.013	25.98	−0.019	134.81
Avoidable variables	0.018	31.84	0.037	74.87	0.006	−43.66
Behavior	−0.001	−1.50	0.000	−0.11	0.000	3.29
BMI	0.000	0.00	0.000	0.00	0.011	−79.78
Income-related	0.019	33.34	0.037	74.98	−0.005	32.83
Residual	−0.001	−1.58	0.000	−0.85	−0.001	8.85
Concentration index	0.057	100.00	0.064	100.00	−0.014	100.00
Horizontal inequity index	0.017		0.036		0.005	

## Data Availability

The data used in this study belong to the Shaanxi Health and Family Planning Commission (SHFPC) and contain the personal information (e.g., name, ID, etc.) of participants. The authors were involved in data collection. Due to the sensitive nature of these data and restrictions imposed by the SHFPC, the authors cannot make these data publicly available. Other researchers who want to use the data may submit requests for data access to the SHFPC at sxwjwwz@126.com.

## Abbreviations

CI	Concentration index
NCD	Non-communicable disease
NHSS	National Health Services Survey
LMICs	Low- and middle-income countries
SES	Socioeconomic Status
95 CI	95% confidence interval
